# Chromosome-level genome assembly of *Nibea coibor* using PacBio HiFi reads and Hi-C technologies

**DOI:** 10.1038/s41597-022-01804-6

**Published:** 2022-11-03

**Authors:** Dinaer Yekefenhazi, Qiwei He, Xiaopeng Wang, Wei Han, Chaowei Song, Wanbo Li

**Affiliations:** Key Laboratory of Healthy Mariculture for the East China Sea, Ministry of Agriculture and Rural Affairs, Jimei University, Xiamen, China

**Keywords:** Data processing, Evolutionary genetics

## Abstract

*Nibea coibor* belongs to Sciaenidae and is distributed in the South China Sea, East China Sea, India and the Philippines. In this study, we sequenced the DNA of a male *Nibea coibor* using PacBio long-read sequencing and generated chromatin interaction data. The genome size of *Nibea coibor* was estimated to be 611.85~633.88 Mb based on k-mer counts generated with Jellyfish. PacBio sequencing produced 29.26 Gb of HiFi reads, and Hifiasm was used to assemble a 627.60 Mb genome with a contig N50 of 10.66 Mb. We further found the canonical telomeric repeats “TTAGGG” to be present at the telomeres of all 24 chromosomes. The completeness of the assembly was estimated to be 98.9% and 97.8% using BUSCO and Merqury, respectively. Using the combination of *ab initio* prediction, protein homology and RNAseq annotation, we identified a total of 21,433 protein-coding genes. Phylogenetic analyses showed that *Nibea coibor* and *Nibea albiflora* are closely related. The results provide an important basis for research on the genetic breeding and genome evolution of *Nibea coibor*.

## Abstract (summary)

**Table:** 

Measurement(s)	Whole-Genome Shotgun Sequencing • transcription profiling assay
Technology Type(s)	single molecular realtime sequencing • RNA sequencing
Sample Characteristic - Organism	Nibea coibor
Sample Characteristic - Location	China

## Background & Summary

**Fig. 1:**
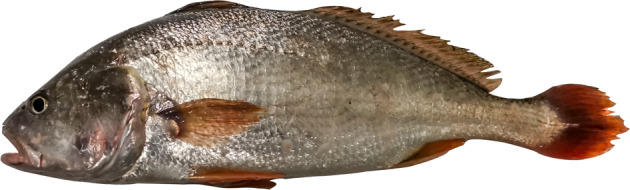
Picture of *Nibea coibor*.

*Nibea coibor* belongs to the family Sciaenidae and is mainly distributed in the South China Sea, East China Sea, India and the Philippines^1^ (Fig. 1). As a fast-growing fish, it is widely cultured along the coast of China and has high nutritional and economic value. Early research on this fish mainly focused on breeding methods and biological characterization. In recent years, studies have focused on feed nutrition^2–6^, growth^7–9^ and development^10–12^. There are reports on the mitochondrial genome in *Nibea coibor*^1,13^; however, the lack of a genome assembly has hindered genetic and evolutionary research on this species.

Recently, single-molecule sequencing^14^ has developed rapidly due to its advantages of long read length, fast speed and high accuracy and has become the mainstream sequencing method for genome assembly. This technology has been successfully adopted in assembling the genomes of fish, such as *Oreochromis mossambicus*^15^, *Acanthopagrus latus*^16^, *Scatophagus argus*^17^ and *Hypophthalmichthys molitrix*^18^. The newly updated high-fidelity (HiFi) sequence reads produced under the circular consensus sequencing (CCS) mode from PacBio achieve a balance between read length and base quality^19^. Some assembly software for processing HiFi reads, including HiCanu^20^, Falcon^21^, and Hifiasm^22^, is available. Among them, Hifiasm^22^ is the latest haplotype-resolved genome assembly algorithm for long HiFi reads. Hifiasm first performs all-versus-all read overlap alignment and then performs three rounds of error correction for sequencing errors by default. The corrected reads were then used to generate overlap alignment again and build a string graph. Hifiasm arbitrarily selects one haplotigs if heterozygous alleles present, and outputs a primary assembly and an alternate assembly. It resolves repetitive sequence information, such as centromeric and telomeric information. Compared with other existing algorithms, Hifiasm^22^ has the advantages of fast assembly speed, high accuracy and continuity. The long high-fidelity sequence reads of the Hifiasm^22^ assembly algorithm, combined with Hi-C^23^ technology, enable assembly of chromosome-level genomes with high quality. However, Hifiasm cannot resolve highly repetitive regions properly^24^.

In this study, we extracted DNA from a male *Nibea coibor* and generated HiFi reads using the PacBio platform. A high-quality contig assembly was produced using Hifiasm. Along with Hi-C data, Juicer and 3D-DNA were used to assemble and generate chromosome-level genomes. Three strategies were then used to annotate the genome. In addition, phylogenetic analyses based on single-copy genes were performed to understand the relationship between *Nibea coibor* and other species. This is the first genome assembly of *Nibea coibor*, which will be helpful to understand the gene structure, function and arrangement of this species, providing a basis for subsequent studies on genetic breeding, evolutionary analysis and germplasm resource conservation.

## Methods

### Library construction and sequencing

Genomic DNA was isolated from the liver and fin of a male *Nibea coibor* using the phenol/chloroform method for long-read and short-read sequencing, respectively. HiFi SMRTbell libraries were prepared using SMRTbell Express Template Prep Kit 2.0 (PacBio, CA, USA). The gDNA was sheared to 15~18 kb with a g-TUBE (Covaris, MA, USA), and DNA damage and fragment ends were repaired using reagents included in Template Prep Kit. SMRTbell hairpin adapters were ligated to the repaired ends, and AMPure PB beads (PacBio, CA, USA) were then used for library concentration and purification. To obtain large-insert SMRTbell libraries for sequencing, SMRTbell templates larger than 15 kb were size-selected with the BluePippin system (SageScience, MA, USA). Sequencing was carried out by Novogene (Beijing, China) using the PacBio Sequel II platform. Subsequently, CCS software (https://github.com/PacificBiosciences/ccs) was used to produce high-precision HiFi reads with quality above Q20, with standard settings of Min passes = 3 and min RQ = 0.99 (Table 1). SMRTbell adapter contamination in the HiFi reads was checked using cutadapt (v2.10)^25^, requiring at least 15 bp of overlap (error rate = 0.1) with adapter sequences. We found that only 284 of 1,919,461 reads contained adapters, and the adapter-contaminated reads were filtered out. Finally, we retained 29.26 Gb of HiFi data, with the longest length, average length and N50 of read length being 39.74, 15.24 and 15.34 kb (Table 2), respectively. The DNA extracted from the fin was sequenced using the Illumina NovaSeq 6000 platform by Novogene (Beijing, China), generating 19.79 Gb raw paired-end reads with 150-bp read length.

**Table 1:** Statistics of different types of sequencing reads.

Type	Sample	Platform	Data (Gb)
CCS	Liver	PacBio Sequel II	110
Hi-C	Liver	Illumina NovaSeq 6000	88.96
DNAseq	Fin	Illumina NovaSeq 6000	19.79
RNAseq	Pooled	Illumina NovaSeq 6000	17.04

**Table 2:** Assembly statistics at the contig level and scaffold level.

Type	Contig (bp)	Scaffold (bp)
Number	314	230
N10	19,501,364	28,157,289
N50	10,661,651	26,221,791
N90	2,170,199	17,275,723
Max length	23,262,851	31,605,326
Total length	627,603,018	627,661,018

Total RNA was extracted from the liver, muscle, testis and ovary tissues from a male and a female using TRIzol Reagent (Invitrogen, MA, USA) according to the manufacturer’s instructions and then pooled with equal molar concentrations for RNA sequencing. Total RNA was selected with oligo (dT) beads and disrupted into short fragments by adding fragmentation buffer. These short fragments were used to synthesize first-strand cDNA using random hexamer primers, followed by synthesis of second-strand cDNA. AMPure XP beads were employed to purify double-stranded cDNA, and EB buffer was used for end-repair and A-tailing. The constructed RNA library was quantified and diluted, and an Agilent 2100 Bioanalyzer system (Agilent Technologies, CA, USA) was employed to assess insert sizes. qPCR was used to accurately quantify the effective concentration of the library. Sequencing of the RNA library was performed using the Illumina NovaSeq 6000 platform (Novogene, Beijing, China) and yielded a total of 17.04 Gb paired-end raw reads, with a Q30 of 93.67% (Table 1).

Hi-C data were generated using liver tissue samples from a male *Nibea coibor*. The Hi-C library was constructed using liver tissue following the protocol described by Belton *et al*.^26^, with some modifications. In brief, tissue was ground and then cross-linked with 4% formaldehyde solution. After quenching the crosslinking reaction and lysis, nuclei were resuspended in NEB buffer and solubilized with dilute SDS, and the 4-cutter restriction enzyme MboI (400 units) was used for digestion. DNA was purified by phenol‒chloroform extraction. The constructed library was paired-end sequenced using the Illumina NovaSeq 6000 platform. The sequenced raw data were filtered to obtain a total of 88.96 Gb of clean data (Table 1), with Q20 = 96.74% and Q30 = 91.82%, which was used to assist chromosome assembly.

### Assembling and genome quality assessment

The genome was assembled using the default parameters of Hifiasm (v0.13.0-R307)^22^. We used HiFi reads without additional data, such as parental data, to generate a primary assembly graph. We precomputed overlaps and reperformed overlapping from the corrected reads and purged haplotig duplications with Hifiasm and carried out three rounds of error correction. The assembled graph yielded 314 contigs with a size of 627.60 Mb. The maximum contig size and N50 were 23.26 and 10.66 Mb (Table 2), respectively.

**Fig. 2:**
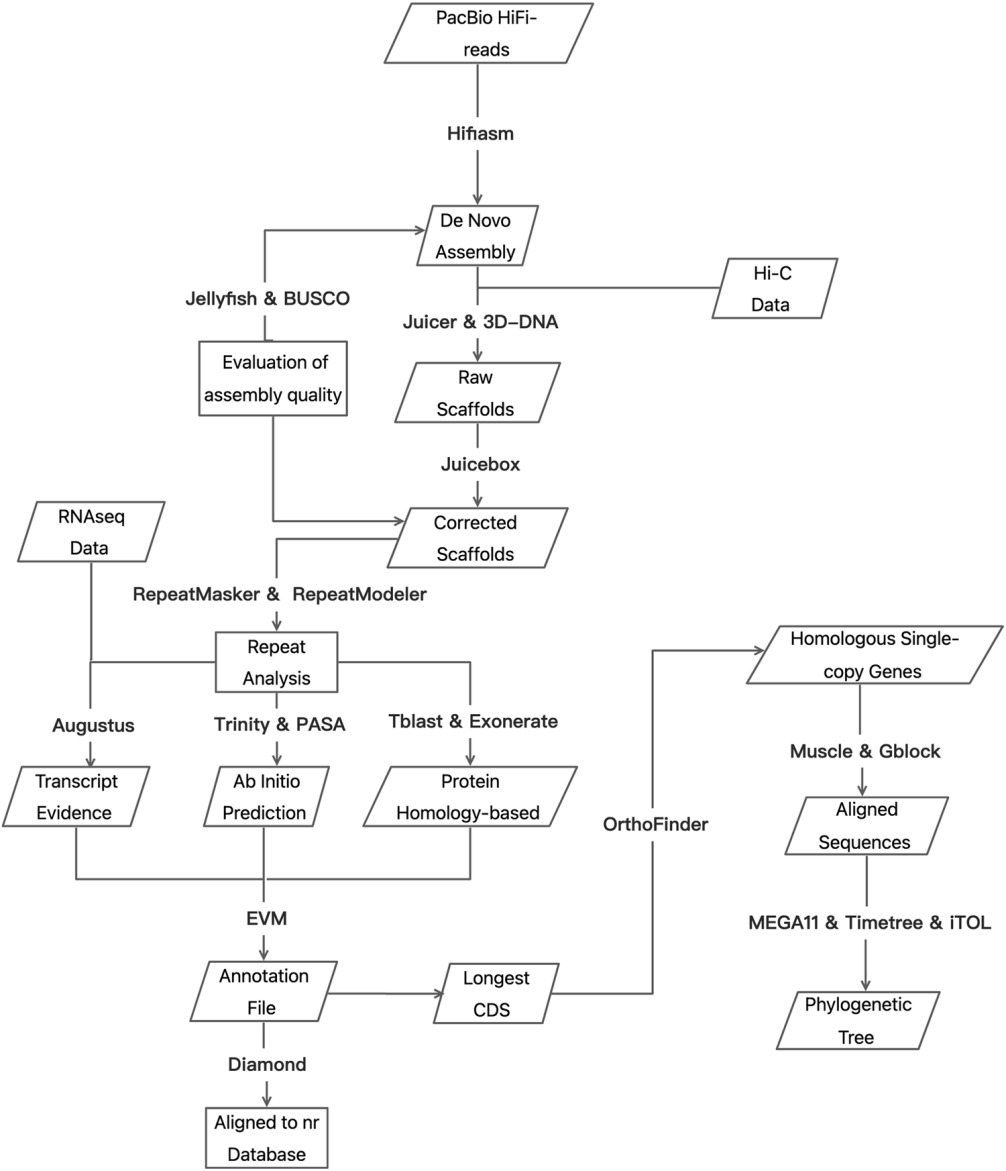
The workflow of genome assembly, annotation and phylogenetics.

**Fig. 3:**
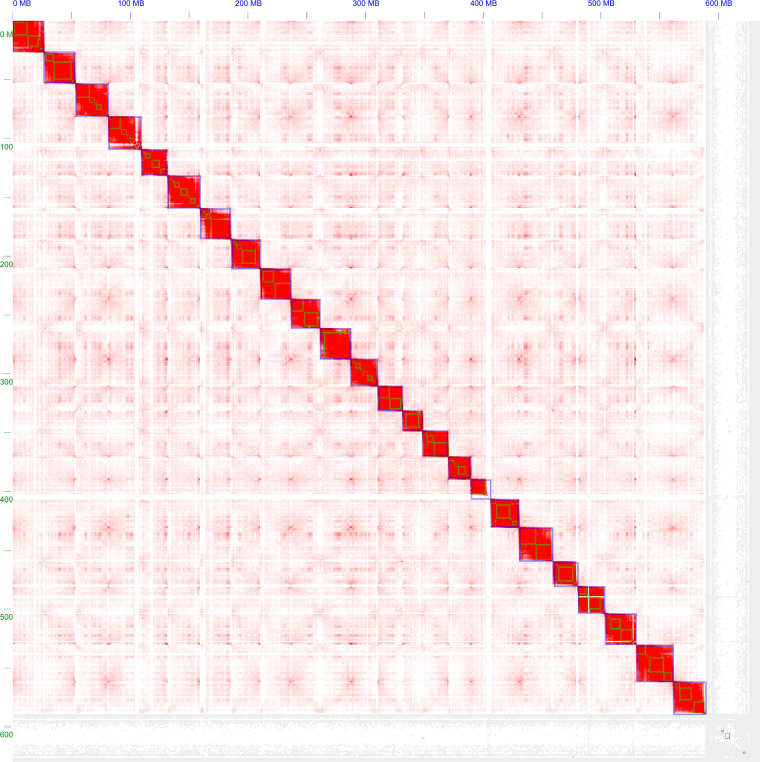
Genome-wide Hi-C heatmap of *Nibea coibor*. The blue squares represent chromosomes and the small green squares inside the blue squares represent contigs that make up the chromosome. The blue squares contained in grey area are shrapnels.

Juicer^27^ (v1.6) combined with 3D-DNA^28^ (v180419) was used for scaffolding. First, HiCUP^29^ (v0.8.1) was used to process the Hi-C data. BWA^30^ (v0.7.17-r1188) was used to index the contig-level genome, and Juicer was then used to create restriction enzyme cutting sites. The processed Hi-C data were further analysed and processed using Juicer (v1.6). In brief, we set the restriction type (S), reference genome file (Z), restriction enzyme cutting site file (Y), and chromosome size file (P). The run-ASM-pipeline.sh script of 3D-DNA was utilized to scaffold a draft reference genome, and an assembly heatmap was generated using 3D-DNA (Fig. 2). Juicerbox^31^ (v1.11.08) was used to manually correct assembly errors (mostly translocations errors), and we ultimately resolved 24 chromosomes (Fig. 3). The run-ASM-pipeline-post-review.sh script of 3D-DNA^28^ was used again to revise the results of the modified file output by Juicerbox, and the “FINAL” assembly was obtained with a total of 230 scaffolds. The maximum scaffold size and N50 size were 31.60 and 26.22 Mb (Table 2), respectively.

In addition, the distribution of telomere repeat sequences in the assembled genome was detected based on vertebrate telomere sequence information^32^ provided by Telomerase Database (http://telomerase.asu.edu/sequences_telomere.html). The results showed that all 24 chromosomes contained telomere repeat sequences, namely, the repeat sequence ‘TTAGGG’ and its reverse complement ‘CCCTAA’, and 14 of them contained a large number of repeat sequences ranging from 14 to 1,365 (Supplementary Fig. 1).

### Genome size and completeness estimation

Jellyfish^33^ (v2.3.0) was used to count the k-mers by setting the k-mer parameters to 19, 23, 27, and 31 (Table 3 and Supplementary Fig. 2), and to obtain the corresponding frequency distributions using the high-coverage short reads. The estimated genome size of *Nibea coibor* ranges from 611.85 Mb (19-mer) to 633.88 Mb (23-mer) (Table 3, Supplementary Fig. 2).

**Table 3:** Estimation of genome size using Jellyfish counts.

K-mer (bp)	19	23	27	31
Total Nod	9,731	9,559	9,357	9,079
Total K-mers	12,237,039,681	11,409,856,779	10,648,458,131	9,944,011,007
Peak	20	18	17	16
Estimated size	611,851,984	633,880,932	626,379,890	621,500,688
Single copy	535,546,028	570,186,037	566,065,646	563,897,168
Proportion	0.88	0.90	0.90	0.91

Benchmarking Universal Single-Copy Orthologues (BUSCO)^34^ (v5.1.2) was also used to assess genome completeness with the actinopteryGIi_ODb10 database (https://busco-data.ezlab.org). A total of 3,640 BUSCO genes were identified, with 3,600 complete genes, 3,552 single-copy genes, 48 multi-copy genes and 29 missing genes accounting for 98.9%, 97.6%, 1.3% and 0.3% of the whole genome, respectively (Table 4). In addition, Merqury^35^ was used to evaluate the QV value and completeness of the genome with both HiFi and Illumina reads. As a result, the completeness of the genome reached 97.8% using both HiFi and Illumina short reads. The QVs were 61.9 and 46.6 estimated with HiFi and Illumina k-mers, respectively. The k-mer spectrum plots generated with Merqury showed no abnormal false duplications in our genome assembly, and the k-mers that appeared only in the assembly, and not in the sequencing reads (implying base errors in the assembly), were trivial (Supplementary Fig. 3**)**.

**Table 4:** Results of BUSCO assessment.

Type	Number	Percentage
Complete	3,600	98.90%
Single-copy	3,552	97.60%
Duplicated	48	1.30%
Fragment	11	0.30%
Missing	29	0.80%
Total	3,640	\

### Repeat‐content identification and annotation

The RepbaseTE library was used to detect repeated sequences in the chromosome-scale genome assembly with the RepeatMasker program^36^ (v4.0.6), and RepeatModeler^37^ (v1.0.9) was used to construct a de novo repeat library. Based on the results, repetitive sequences comprise 11.49 Mb, accounting for 18.31% of the assembled genome. Among the repeat elements, short interspersed nuclear elements (SINEs) account for 0.58% of genome size and long interspersed nuclear elements (LINEs) for 1.79%. Long terminal repeats (LTRs) and DNA elements account for 1.37% and 3.11%, respectively. The small RNA content is 0.46%, and satellites and simple repeats account for 0.15% and 2.72%, respectively.

A combined strategy of *ab initio*, transcript evidence and protein homology-based gene prediction methods was performed for gene annotation. The pooled RNAseq clean data were assembled in two ways, i.e., transcript assembly relied on the reference genome and de novo assembly using Trinity software^38^ (v2.4.0), and open reading frames (ORFs) were identified using PASA^39^ (v2.1.0). Augustus^40^ (v3.2.3) was employed to perform *ab initio* gene prediction using known genes of zebrafish and the transcripts assembled from RNAseq. The optimal parameters were obtained after two rounds of model training. Tblastn^41^ was used to align the protein sequences of *Nibea coibor* and 9 other species, including *Cynoglossus semilaevis, Danio rerio* (zebrafish), *Takifugu rubripe* (pufferfish), *Dicentrarchus labrax* (European seabass), *Gasterosteus aculeatus* (three‐spined stickleback)*, Larimichthys crocea* (large yellow croaker), *Lates calcarifer, Oreochromis niloticus* and *Oryzias latipes* (medaka), for homology-based gene prediction. Exonerate^42^ (v2.2.0) was used to accurately locate splice sites and exons of aligned sequences. Genes with coding regions less than 150 bp were then discarded, and the results of the three gene prediction models were weighted and evaluated by Evidence Modeller (EVM)^43^ (v1.1.1) to produce a comprehensive and reliable gene structure containing coding regions and alternative splice sites. All predicted genes were aligned to the NCBI nonredundant protein (nr) database and functionally annotated using blastp^44^. Ultimately, 21,433 genes were predicted, including 14,633 non-alternatively spliced genes and 6,800 alternatively spliced genes. Of these genes, 19,859 genes were annotated in the NCBI nr database.

### Phylogenetic analysis

**Fig. 4:**
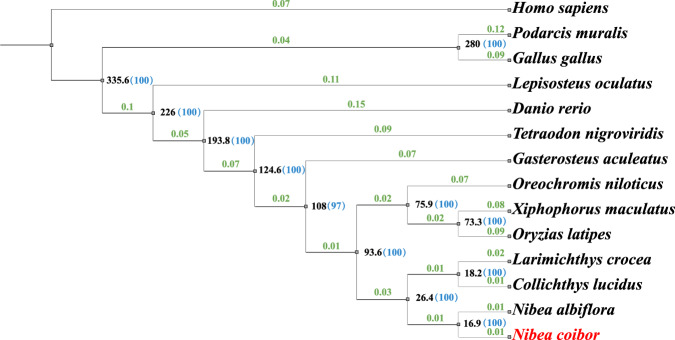
The phylogenetic analysis of *Nibea coibor* and other 13 species. The numbers in green on the branches indicate average number of nucleotide substitutions per site (the length of the branches not accurately represent the substitution rate), the numbers in black near the nodes indicate the divergence time (million years ago, MYA), and the blue numbers inside the brackets are bootstrap values.

Coding sequences (CDSs) of 13 species, including *Homo sapiens, Podarcis_muralis, Gallus, Lepisosteus oculatus, Danio rerio, Larimichthys crocea, Xiphophorus maculatus, Tetraodon nigroviridis, Oreochromis niloticus, Oryzias latipes, Gasterosteus aculeatus, Nibea albiflora*^45^ and *Collichthys lucidus*^46^, were retrieved from Ensmbl or NCBI databases. The longest CDS of each gene for each species was extracted, and homology analysis was performed using OrthoFinder^47^ (v2.5.4) with default settings. A total of 333,401 genes were identified in the 14 species, including 1,876 homologous single-copy genes. These homologous single-copy genes were compared using the “-align” parameter of Muscle^48^ (v5.1). Gblock^49,50^ (v0.19b) was employed to extract conserved sequences in comparison results with the parameter “-b4 = 5 -b5 = h -t = d -e = 0.2”, and Seqkit^51^ (v2.2.0) was used to merge the results. The phylogenetic tree was constructed via MEGA11^52^, *with H. sapiens* as the outgroup, and Timetree^53^ was used to estimate the divergence time of other vertebrates based on the divergence time of chickens and lizards (280 MYA). The evolutionary tree was visualized using iTOL^54^ (https://itol.embl.de/). According to our phylogenetic tree (Fig. 4), we observed that *Nibea coibor* is evolutionarily closer to *Nibea albiflora*, which also belongs to *Nibea*, with a divergence time of 16.9 MYA. In addition, the two species have a common ancestor with *Larimichthys crocea* and *Collichthys lucidus*, which belong to the same family Sciaenidae, and the divergence time of the two clades is 26.4 MYA.

The complete sequence of the mitochondrion (GenBank ID: CM041792.1) of *Nibea coibor* is included in our assembly. The mitochondrion contains 13 protein-coding genes, 22 tRNA and 2 rRNA genes annotated with MITOS Web Server^55^ (http://mitos.bioinf.uni-leipzig.de/index.py). The longest mitochondrial CDSs of the above 13 species and *Nibea coibor* were compared using Clustal Omega (v1.2.4)^56^. The phylogenetic tree based on mitochondrial sequences was constructed with IQ-TREE (v1.6.12)^57,58^ and suggests that *Nibea coibor* is closer to *Nibea albiflora*, *Larimichthys crocea* and *Collichthys lucidus* (Supplementary Fig. 4).

## Data Records

The genomic Illumina sequencing data were deposited in the SRA at NCBI SRR19088065^59^.

The genomic PacBio sequencing data were deposited in the SRA at NCBI SRR19088064^60^.

The transcriptomic sequencing data were deposited in the SRA at NCBI SRR19088063^61^.

The Hi-C sequencing data were deposited in the SRA at NCBI SRR19088062^62^.

The final chromosome assembly was deposited in GenBank at NCBI JALLKU000000000^63^.

The genome annotation file is available in figshare^64^.

## Technical Validation

The DNA extracted for paired-end sequencing was checked using agarose gel electrophoresis, and the concentration of the DNA was determined using a Qubit Fluorometer (Thermo Fisher Scientific, USA).

The DNA extracted for PacBio sequencing was also checked by agarose gel electrophoresis, showing a main band above 30 kb. The concentration of DNA was determined using a Qubit Fluorometer (Thermo Fisher Scientific, USA), and absorbance was 1.802 at 260/280 using a NanoDrop ND-1000 spectrophotometer (LabTech, USA).

For RNA-seq, total RNA was extracted using TRIzol reagent (Invitrogen, MA, USA) following the manufacturer’s protocol. RNA integrity was evaluated using an Agilent 2100 Bioanalyzer (Agilent Technologies, CA, USA). The sample used in our study had an RNA integrity number (RIN) larger than 8.5.

We generated 89.62 Gb of Hi-C raw reads, and the effective rate was 99.26%. The Q20 and Q30 base qualities of the Hi-C reads were 96.74% and 91.82%, respectively.

## Supplementary information

Supplementary figures

## Data Availability

No specific code was used in this study. The data analyses used standard bioinformatic tools specified in the methods.
