# Supplementary material for: Chromosome-level genome assembly of Nibea coibor using PacBio HiFi reads and Hi-C technologies
Source: Sci Data. 2022 Nov 3;9:670. doi: 10.1038/s41597-022-01804-6 (PMC9633807; doi:10.1038/s41597-022-01804-6)
Supplement: Supplementary file 1 — Supplementary figures [file 41597_2022_1804_MOESM1_ESM.pdf]

Supplementary Figures

Supplementary Figure 1 2

Supplementary Figure 2 3

Supplementary Figure 3 4

Supplementary Figure 4 5

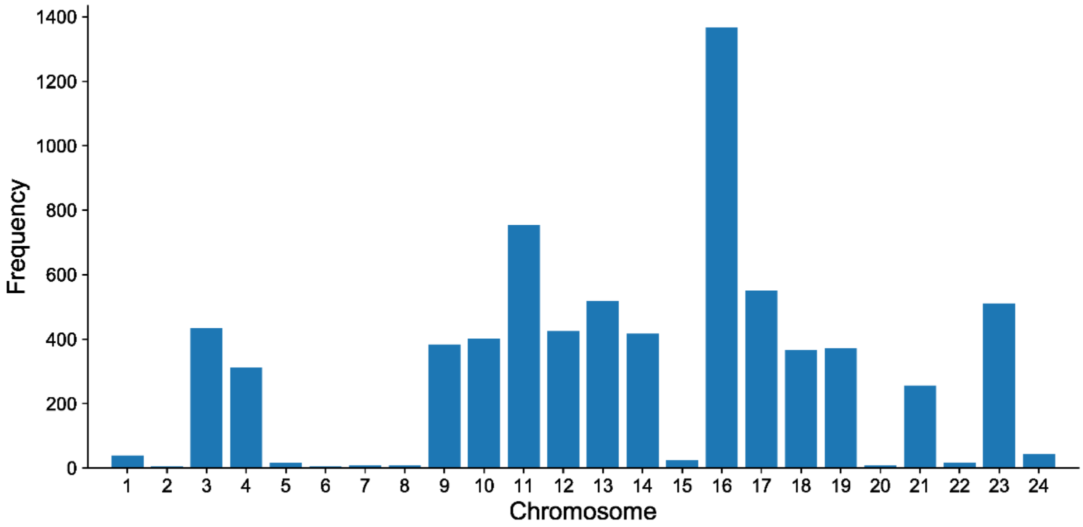

Supplementary Figure 1. Frequency of telomeric repeats on 24 chromosomes.

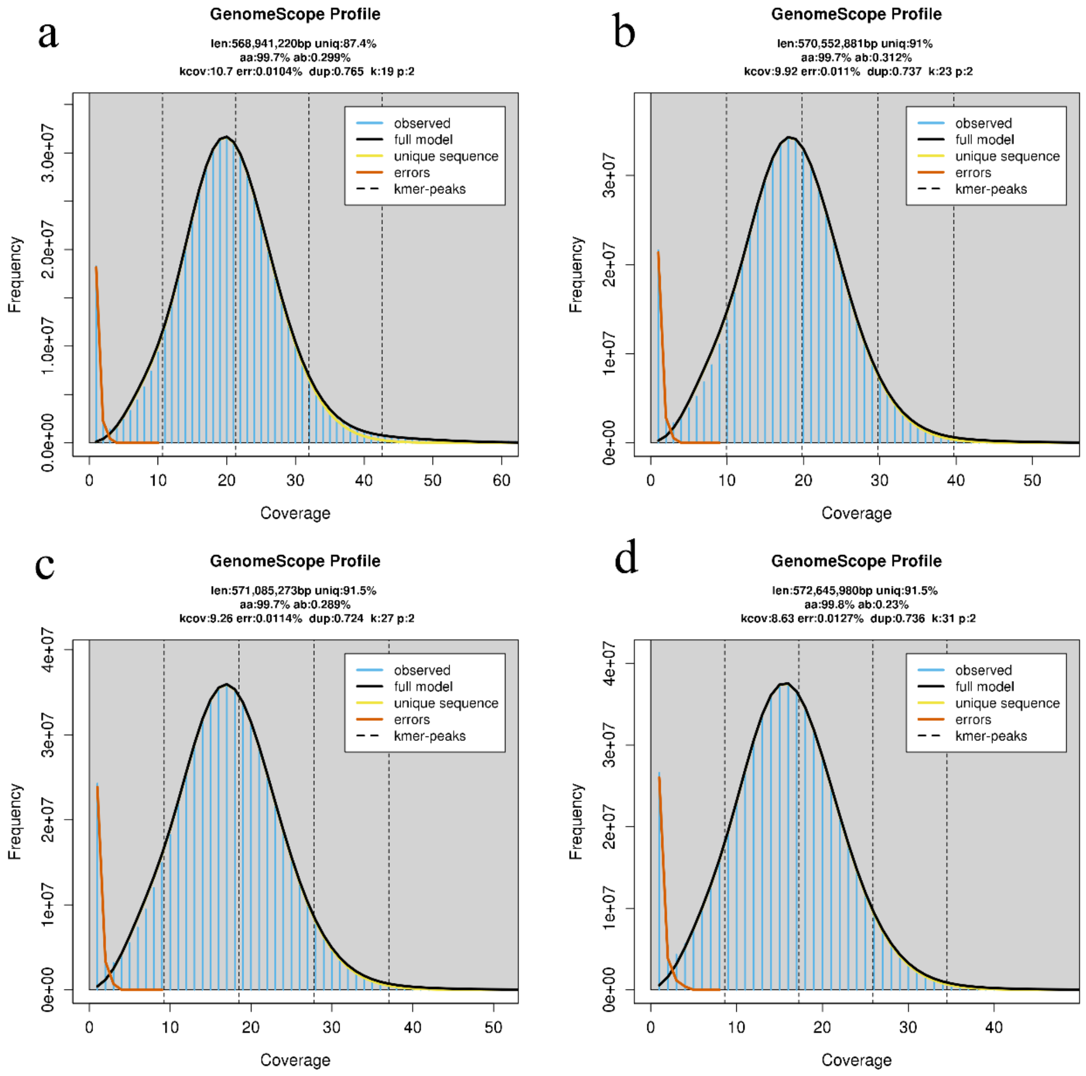

Supplementary Figure 2. K-mer distribution of whole-genome sequencing reads of *Nibea coibor*. Figures a, b, c, and d correspond to k-mer values of 19, 23, 27, and 31, respectively.

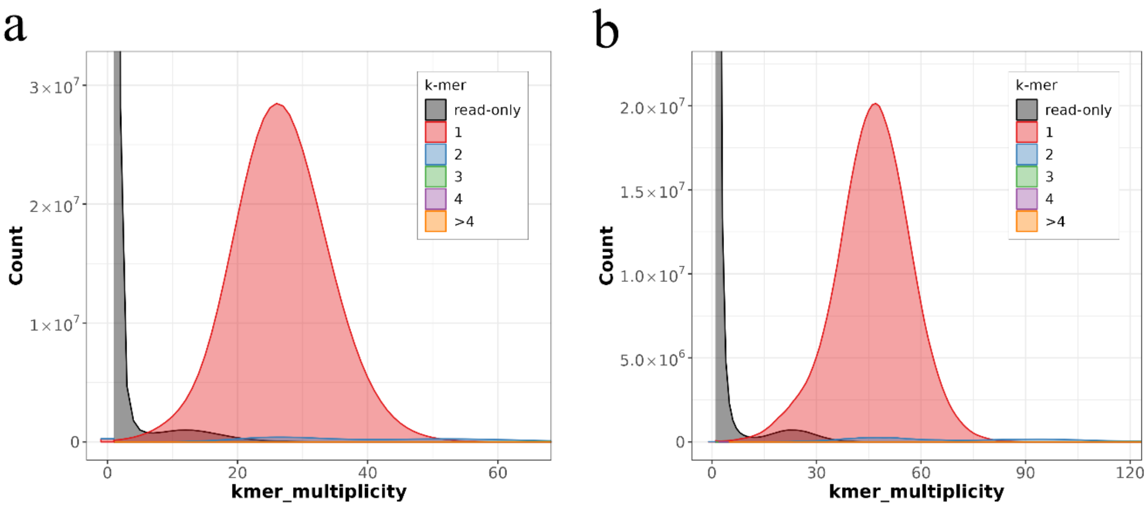

Supplementary Figure 3. Merqury copy number spectrum plots for the assembly of *Nibea coibor* generated with Illumina (a) and PacBio (b) reads. The majority of k-mers appear under the "1-copy" peak because the assembly was not haplotype resolved.

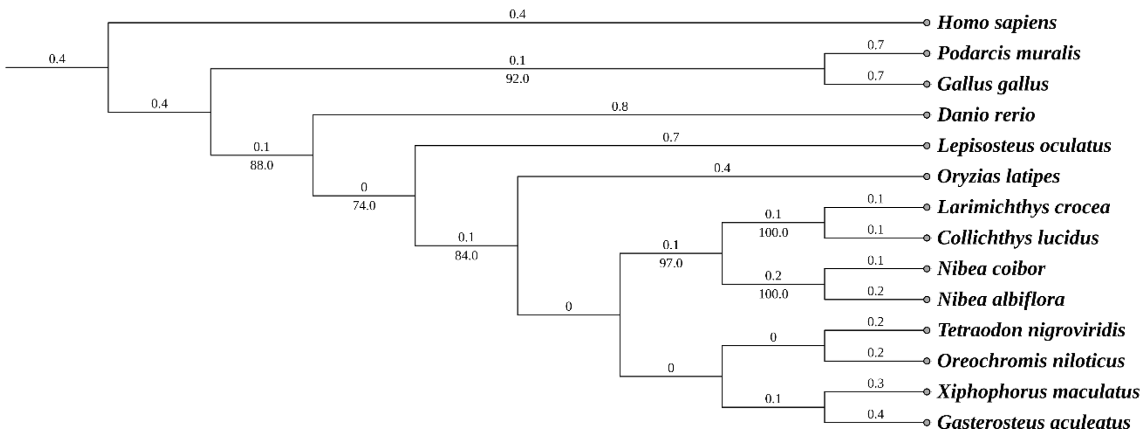

Supplementary Figure 4. The mtDNA tree of *Nibea coibor* and 13 other species. The numbers on the branches are branch length, and bootstrap values are noted below the branch.
